# Increased Tumor Necrosis Factor (TNF)-α and Its Promoter Polymorphisms Correlate with Disease Progression and Higher Susceptibility towards Vitiligo

**DOI:** 10.1371/journal.pone.0052298

**Published:** 2012-12-20

**Authors:** Naresh C. Laddha, Mitesh Dwivedi, Rasheedunnisa Begum

**Affiliations:** Department of Biochemistry, Faculty of Science, The Maharaja Sayajirao University of Baroda, Vadodara, Gujarat, India; University of Hyderabad, India

## Abstract

Tumor Necrosis Factor (TNF)-α, is a paracrine inhibitor of melanocytes, which plays a critical role in the pathogenesis of several autoimmune diseases including vitiligo, as abnormal immune responses have frequently been observed in vitiligo patients. Moreover, vitiligo patients show higher lesion levels of TNF-α. Genetic polymorphisms in the promoter region of *TNF-α* are involved in the regulation of its expression. The present study explores *TNF*-α promoter polymorphisms and correlates them with *TNF-α* transcript and protein levels in vitiligo patients and controls of Gujarat along with its effect on disease onset and progression. PCR-RFLP technique was used for genotyping of these polymorphisms in 977 vitiligo patients and 990 controls. TNF-α transcript and protein levels were measured by Real time PCR and ELISA respectively. The genotype and allele frequencies for the investigated polymorphisms were significantly associated with vitiligo patients. The study revealed significant increase in *TNF*-α transcript and protein levels in vitiligo patients compared to controls. In particular, haplotypes: AATCC, AACCT, AGTCT, GATCT, GATCC and AGCCT were found to increase the TNF-α levels in vitiligo patients. Analysis of TNF-α levels based on the gender and disease progression suggests that female patients and patients with active vitiligo had higher levels of TNF-α. Also, the TNF-α levels were high in patients with generalized vitiligo as compared to localized vitiligo. Age of onset analysis of the disease suggests that the haplotypes: AACAT, AACCT, AATCC and AATCT had a profound effect in the early onset of the disease. Moreover, the analysis suggests that female patients had an early onset of vitiligo. Overall, our results suggest that *TNF*-α promoter polymorphisms may be genetic risk factors for susceptibility and progression of the disease. The up-regulation of *TNF*-α transcript and protein levels in individuals with susceptible haplotypes advocates the crucial role of TNF-α in autoimmune pathogenesis of vitiligo.

## Introduction

Vitiligo is an acquired, non-contagious disease in which progressive, patchy, multifocal loss of pigmentation of skin, overlying hair, and often mucous membranes results from loss of melanocytes from the involved areas [1]. It affects 0.2–1% of the world population [2]. In India, the incidence of vitiligo is found to be 0.5% [3]. It is associated with increased risk of several other autoimmune diseases such as: autoimmune thyroid disease (Graves’ disease and autoimmune hypothyroidism), rheumatoid arthritis, psoriasis, adult-onset autoimmune diabetes mellitus, pernicious anemia, Addison’s disease, and systemic lupus erythematosus [4],[5]. The autoimmune destruction of melanocytes can be explained by the abnormalities in both humoral and cell-mediated immunity [6],[7]. The autoimmune hypothesis gains further support from immunotherapy studies of melanoma patients [8].

Vitiligo is a polygenic disease; however, recent genome-wide association studies (GWAS), have identified generalized vitiligo susceptibility genes which almost universally involve immune regulation and immune targeting of melanocytes, that have led to the general consensus that generalized vitiligo is a primary autoimmune disease, though the biological triggers of the autoimmune process remain unknown [9]. Several candidate genes have been tested for genetic association with generalized vitiligo, including the *MHC*, *ACE*, *CAT*, *CTLA*-4, *COMT*, *ESR*, *GCH*1, *MBL*2, *PTPN*22, and *VDR* [10],[2]. Most of these studies reported significant associations, although some yielded only marginal significance and several were not replicated by subsequent studies. Recently, a number of genes which play a role in vitiligo susceptibility, including *HLA*, *PTPN*22, *NALP*1, *XBP*1, *FOXP*1, *IL*2RA have been tested for genetic association with vitiligo [11].

Cytokines are important mediators of immunity and their response due to imbalance or deficiency in the cytokine network may largely determine autoimmune disease susceptibility and severity. Tumor necrosis factor (TNF)-α is a multifunctional, proinflammatory cytokine which plays an important role in several autoimmune diseases like rheumatoid arthritis, pernicious anemia, diabetes mellitus etc.

TNF-α plays important role in apoptosis through activation of the receptor-mediated apoptosis pathway in numerous cell types [12]. It is produced by many different cell types, including activated T cells, fibroblasts, adipocytes, smooth muscle cells and keratinocytes. In the epidermal melanin unit of epidermis, a melanocyte is in close interaction with ∼32 keratinocytes. The keratinocytes synthesize cytokines, such as TNF-α, interleukin (IL)-1a, IL-6, and transforming growth factor-β (TGF-β), which are paracrine inhibitors of human melanocyte proliferation and melanogenesis. TNF-α also affects the apoptotic pathway of melanocytes and its level may play an important role in vitiligo pathogenesis. Moreover, TNF-α can inhibit melanocyte stem cell differentiation [13].

*TNF* gene locus is located within the Class III region of the human major histocompatibility complex (MHC) on chromosome 6 (6p21.31) spanning about 3 kb and contains 4 exons. Regulation of TNF-α production occurs at both the transcriptional and post-transcriptional levels, with regulatory sequences within the 5′ end of the gene controlling the rate of transcription [14]. Several single-nucleotide polymorphisms (SNPs) have been identified in the human *TNF*-α gene promoter region having the potential to cause structural changes within regulatory sites that could affect the function or regulation of TNF-α production. The location of its gene within major histocompatibility complex and biological activities has raised the possibility that polymorphisms within this locus may contribute to the pathogenesis of wide range of autoimmune and infectious diseases.

The promoter polymorphisms at positions: −238, −308, −857, and −1031 may lead to a higher rate of *TNF*-α gene transcription whereas −863 leads to decrease in the transcription. These polymorphisms combined could contribute to the autoimmune process making it an ideal candidate for the development of vitiligo.

In the present study, we have made an attempt to understand the role of TNF-α in vitiligo pathogenesis. Hence, the aims of this study were: i.) to determine whether the promoter polymorphisms of *TNF*-α [−238 (G/A; rs361525), −308 (G/A; rs1800629), −857 (C/T; rs1799724), −863 (C/A; rs1800630) and −1031 (T/C; rs1799964)] are associated with vitiligo susceptibility and modulate *TNF*-α transcript and protein levels. ii.) to measure and compare *TNF*-α transcript and protein levels in patients with vitiligo and in unaffected controls. iii.) to correlate *TNF*-α polymorphisms/levels with onset and progression of the disease.

## Materials and Methods

### Study Subjects

The study group included 977 vitiligo patients [733 generalized (including acrofacial vitiligo and vitiligo universalis) and 244 localized vitiligo cases] comprised of 451 males and 526 females who referred to S.S.G. Hospital, Vadodara and Civil Hospital, Ahmedabad, Gujarat, India (Table S1). The diagnosis of vitiligo was clinically based on the presence of depigmented patches on the skin and patients had no other associated autoimmune diseases. A total of 990 ethnically and sex-matched unaffected individuals (447 males and 543 females) were included as controls in the study (). None of the healthy individuals or their relatives had any evidence of vitiligo and any other autoimmune disease.

The study plan was approved by the Institutional Ethical Committee for Human Research (IECHR), Faculty of Science, The Maharaja Sayajirao University of Baroda, Vadodara, Gujarat, India. The importance of the study was explained to all participants and written consent was obtained from all patients and controls.

### Genotyping of *TNF*-α Promoter Polymorphisms

Five ml. venous blood was collected from the patients and healthy subjects in K_3_EDTA coated (Greiner Bio-One, North America Inc., North Carolina, USA) tubes. Genomic DNA was extracted from whole blood using ‘whole blood DNA extraction kit’ (Bangalore Genei, Bangalore, India) according to the manufacturer’s instructions. After extraction, concentration and purity of DNA was estimated spectrophotometrically, quality of DNA was also determined on 0.8% agarose gel electrophoresis and DNA was stored at −20°C until further analyses.

Polymerase chain reaction–restriction fragment length polymorphism (PCR-RFLP) was used to genotype all five promoter polymorphisms of *TNF-α* gene (Figure S1). The primers used for genotyping are mentioned in Table S2. The reaction mixture of the total volume of 20 µL included 5 µL (100 ng) of genomic DNA, 10 µL nuclease-free H_2_O, 2.0 µL 10× PCR buffer, 2 µL 2 mM dNTPs (SIGMA Chemical Co, St. Louis, Missouri, USA), 0.3 µL of 10 µM corresponding forward and reverse primers (Eurofins, Bangalore, India), and 0.3 µL (5 U/µL) Taq Polymerase (Bangalore Genei, Bangalore, India). Amplification was performed using a PTC-100 thermal cycler (MJ Research, Inc., Watertown, Massachusetts, USA) according to the protocol: 95°C for 10 min. followed by 30 cycles of 95°C for 15 sec., primer dependent annealing (Table S2) for 30 sec., and 72°C for 30 sec. The amplified products were checked by electrophoresis on a 2.0% agarose gel stained with ethidium bromide.

Restriction enzymes (New England Biolabs, Beverly, MA) used were: *Bam*HI, *Nco*I, *Tai*I and *Bbs*I for digesting amplicons of −238 G/A, −308 G/A, −857 C/T, −863 C/A and −1031 T/C of *TNF-α* gene (Table S2). 5 µL of the amplified products were digested with 5 U of the corresponding restriction enzyme in a total reaction volume of 25 µL as per the manufacturer’s instruction. The digestion products with 100 base pair DNA ladder (Bioron, Ludwigshafen am Rhein, Germany) were resolved on 3.5% agarose gels or 15% polyacrylamide gels stained with ethidium bromide and visualized under UV transilluminator.

More than 10% of the samples were randomly selected for confirmation and the results were 100% concordant (analysis of the chosen samples was repeated by two researchers independently) and also confirmed by sequencing.

### Determination of *TNF*-α and *GAPDH* mRNA Expression

#### RNA extraction and cDNA synthesis

Total RNA from whole blood was isolated and purified using Ribopure™- blood Kit (Ambion inc. Texas, USA) following the manufacturer’s protocol. RNA integrity was verified by 1.5% agarose gel electrophoresis/ethidium bromide staining and O.D. 260/280 absorbance ratio >1.95. RNA was treated with DNase I (Ambion inc. Texas, USA) before cDNA synthesis to avoid DNA contamination. One microgram of total RNA was used to prepare cDNA. cDNA synthesis was performed using the RevertAid First Strand cDNA Synthesis Kit (Fermentase, Vilnius, Lithuania) according to the manufacturer’s instructions in the MJ Research Thermal Cycler (Model PTC-200, Watertown, MA, USA).

#### Real-time PCR

The expression of *TNF-α* and *GAPDH* transcripts were measured by real-time PCR using gene specific primers (Eurofins, Bangalore, India) as shown in Table S2. Expression of *GAPDH* gene was used as a reference. Real-time PCR was performed in duplicates in 20 µl volume using LightCycler®480 SYBR Green I Master (Roche Diagnostics GmbH, Mannheim, Germany) following the manufacturer’s instructions and carried out in the LightCycler®480 Real-Time PCR (Roche Diagnostics GmbH, Mannheim, Germany). The thermal cycling conditions included an initial activation step at 95°C for 10 min., followed by 45 cycles of denaturation, annealing and amplification (95°C for 10 sec., 63°C for 30 sec., 72°C for 30 sec.). The fluorescence data collection was performed during the extension step. At the end of the amplification phase a melting curve analysis was carried out on the product formed. The value of Cp was determined by the first cycle number at which fluorescence was greater than the set threshold value.

### Estimation of Serum TNF-α Levels by Enzyme-linked Immunosorbent Assay

Serum levels of TNF*-α* in patients with vitiligo and controls were measured by enzyme-linked immunosorbent assay (ELISA) using the Immunotech Human TNF*-α* ELISA kit (Immunotech SAS, Marseille Cedex 9, France) as per the manufacturer’s protocol.

### Statistical Analyses

Evaluation of the Hardy-Weinberg equilibrium (HWE) was performed for both the polymorphisms in patients and controls by comparing the observed and expected frequencies of the genotypes using chi-squared analysis. The distribution of the genotypes and allele frequencies of *TNF-α* promoter polymorphisms for patients and control subjects were compared using the chi-squared test with 3×2 and 2×2 contingency tables respectively using Prism 4 software (Graphpad software Inc; San Diego CA, USA, 2003). *p*-values less than 0.01 were considered as statistically significant due to Bonferroni’s correction for multiple testing. Odds ratio (OR) with respective confidence interval (95% CI) for disease susceptibility was also calculated.

Haplotype analysis was carried out using http://analysis.bio-x.cn/myAnalysis.php [15]. The linkage disequilibrium (LD) coefficients D’ = D/Dmax and r^2^-values for the pair of the most common alleles at each site were estimated using the Haploview programe version 4.1 [16]. Relative gene expression of *TNF-α* and serum TNF*-α* levels in patient and control groups was plotted and analyzed by nonparametric unpaired t-test using Prism 4 software (Graphpad software Inc; San Diego CA, USA, 2003). The statistical power of detection of the association with the disease at the 0.05 level of significance was determined by using the G* Power software [17].

## Results

### Association of *TNF*-α Promoter Polymorphisms with Generalized Vitiligo

The genotype and allele frequencies of the investigated *TNF*-α promoter polymorphisms in 733 generalized vitiligo patients and 990 controls are summarized in Table 1. The distribution of genotype frequencies for all the polymorphisms investigated was consistent with Hardy-Weinberg expectations in both patient and control groups (*p*>0·05).

**Table 1:** Association study for *TNF*-α promoter polymorphisms in patients with generalized vitiligo from Gujarat.

SNP	Genotype or allele	Generalized VitiligoPatients (Freq.)	Controls (Freq.)	*p* forAssociation	*p* for HWE	Odds ratio (95% CI)
**rs361525**(−238; G/A)	GenotypeGGGAAAAlleleGA	(n = 729)250 (0.34)339 (0.47)140 (0.19)839 (0.58)619 (0.42)	(n = 990)798 (0.81)178 (0.18)14 (0.01)1774 (0.90)206 (0.10)	<0.0001<0.0001	0.192(P)0.263(C)	6.35 (5.320–7.590)
**rs1800629**(−308; G/A)	GenotypeGGGAAAAlleleGA	(n = 728)317 (0.44)311 (0.43)100 (0.13)945 (0.65)511 (0.35)	(n = 981)780 (0.80)184 (0.19)17 (0.01)1744 (0.89)218 (0.11)	<0.0001<0.0001	0.093(P)0.114(C)	4.326 (3.623–5.165)
**rs1799724**(−857; C/T)	GenotypeCCCTTTAlleleCT	(n = 728)249 (0.34)347 (0.48)132 (0.18)845 (0.58)611 (0.42)	(n = 984)563 (0.57)352 (0.36)69 (0.07)1478 (0.75)490 (0.25)	<0.0001<0.0001	0.563(P)0.173(C)	2.181 (1.885–2.524)
**rs1800630**(−863; C/A)	GenotypeCCCAAAAlleleCA	(n = 728)365 (0.50)287 (0.40)76 (0.10)1017 (0.70)439 (0.30)	(n = 984)698 (0.71)253 (0.26)33 (0.03)1649 (0.84)319 (0.16)	<0.0001<0.0001	0.084(P)0.094(C)	2.231 (1.894–2.629)
**rs1799964**(−1031; T/C)	GenotypeTTTCCCAlleleTC	(n = 733)354 (0.48)295 (0.40)84 (0.12)1003 (0.68)463 (0.32)	(n = 989)653 (0.66)295 (0.30)41 (0.04)1601 (0.81)377 (0.19)	<0.0001<0.0001	0.063(P)0.296(C)	1.960 (1.675–2.294)

‘n’ represents number of Patients/Controls,

HWE refers to Hardy-Weinberg Equilibrium,

CI refers to Confidence Interval,

(P) refers to Patients and (C) refers to Controls,

Statistical significance was considered at *p* value ≤0.01 due to Bonferroni’s correction for multiple testing.

The five promoter polymorphisms of *TNF*-α were found to be in significant association with generalized vitiligo patients (*p*<0.0001) when genotypes were compared using chi-squared test-3×2 contingency table with Bonferroni’s correction for multiple testing (Table 1). Also, there was significant difference in allele frequencies of these polymorphisms between generalized patients and controls when compared with 2×2 contingency table (*p*<0.0001) (Table 1). Interestingly, −238A and −308A alleles were found to increase the risk of generalized vitiligo by 6.35 and 4.326 fold respectively [odds ratio (OR) = 6.35; 95% confidence interval (CI) = 5.320–7.590; OR = 4.326; 95% CI = 3.623–5.165] (Table 1). However, −857T, −863A and −1031C alleles were found to increase the risk of generalized vitiligo by 2.181, 2.231 and 1.96 fold respectively (OR = 2.181, 95% CI = 1.885–2.524; OR = 2.231, 95% CI = 1.894–2.629; OR = 1.960, 95% CI = 1.675–2.294) (Table 1). This study has 95.0% statistical power for the effect size 0.08 to detect association of *TNF*-α promoter polymorphisms at *p*<0.05 in generalized vitiligo patients and control population.

### Association of *TNF*-α Promoter Polymorphisms with Localized Vitiligo

The genotype and allele frequencies of the investigated *TNF*-α promoter polymorphisms in 244 localized vitiligo patients and 990 controls are summarized in Table 2. The distribution of genotype frequencies for all the polymorphisms investigated was consistent with Hardy-Weinberg expectations in both patient and control groups (*p*>0·05) except for −238G/A and −863C/A in patients (*p* = 0.0001 and *p* = 0.014 respectively).

**Table 2:** Association study for *TNF*-α promoter polymorphisms in patients with localized vitiligo from Gujarat.

SNP	Genotype or allele	Localized VitiligoPatients (Freq.)	Controls (Freq.)	*p* forAssociation	*p* for HWE	Odds ratio (95% CI)
**rs361525**(−238; G/A)	GenotypeGGGAAAAlleleGA	(n = 241)75 (0.31)91 (0.38)75 (0.31)241 (0.50)241 (0.50)	(n = 990)798 (0.81)178 (0.18)14(0.01)1774(0.90)206 (0.10)	<0.0001<0.0001	0.0001(P)0.263 (C)	0.116 (0.092–0.146)
**rs1800629**(−308; G/A)	GenotypeGGGAAAAlleleGA	(n = 241)79 (0.33)125 (0.52)37 (0.15)283 (0.59)199 (0.41)	(n = 981)780 (0.80)184 (0.19)17 (0.01)1744 (0.89)218 (0.11)	<0.0001<0.0001	0.278(P)0.114(C)	0.178 (0.141–0.224)
**rs1799724**(−857; C/T)	GenotypeCCCTTTAlleleCT	(n = 241)86 (0.36)117 (0.49)38 (0.15)289 (0.60)193 (0.40)	(n = 984)563 (0.57)352 (0.36)69 (0.07)1478 (0.75)490 (0.25)	<0.0001<0.0001	0.864(P)0.173(C)	0.496 (0.403–0.612)
**rs1800630**(−863; C/A)	GenotypeCCCAAAAlleleCA	(n = 242)137 (0.57)80 (0.33)25 (0.10)354 (0.73)130 (0.27)	(n = 984)698 (0.71)253 (0.26)33 (0.03)1649 (0.84)319 (0.16)	<0.0001<0.0001	0.014(P)0.094(C)	0.527 (0.417–0.666)
**rs1799964**(−1031; T/C)	GenotypeTTTCCCAlleleTC	(n = 244)112 (0.46)104 (0.43)28 (0.11)328 (0.67)160 (0.33)	(n = 989)653 (0.66)295 (0.30)41 (0.04)1601 (0.81)377 (0.19)	<0.0001<0.0001	0.607(P)0.296(C)	0.483 (0.388–0.601)

‘n’ represents number of Patients/Controls,

HWE refers to Hardy-Weinberg Equilibrium,

CI refers to Confidence Interval,

(P) refers to Patients and (C) refers to Controls,

Statistical significance was considered at *p* value ≤0.01 due to Bonferroni’s correction for multiple testing.

The five promoter polymorphisms of *TNF*-α were found to be in significant association with localized vitiligo patients (*p*<0.0001) when genotypes were compared using chi-squared test-3×2 contingency table with Bonferroni’s correction for multiple testing (Table 1). Also, there was significant difference in allele frequencies of these polymorphisms between localized patients and controls when compared with 2×2 contingency table (*p*<0.0001) (Table 1). Although, all five promoter polymorphisms of *TNF*-α were found to be associated with localized vitiligo patients none of the susceptible alleles of these polymorphisms were found to be a risk for localized vitiligo as suggested by the odds ratio (Table 2). This study has 88.0% statistical power for the effect size 0.08 to detect association of *TNF*-α promoter polymorphisms at *p*<0.05 in localized vitiligo patients and control population.

### Linkage Disequilibrium (LD) and Haplotype Analyses of *TNF*-α Promoter Polymorphisms

The LD analysis revealed that the five promoter polymorphisms investigated in the *TNF*-α gene were in low to moderate LD association in both generalized as well as localized vitiligo patients (Figure S2A & S2B). In particular, −238G/A and −308G/A polymorphisms were in moderate LD association with D’ = 0.485 and 0.484 in generalized and localized vitiligo patients respectively (Tables S3 & S4).

A haplotype evaluation of the five polymorphic sites was performed and the estimated frequencies of the haplotypes were differed significantly between generalized vitiligo patients and controls (global *p*<0.0001). Also, localized vitiligo patients exhibited significantly different frequencies of haplotypes as compared to controls (global *p*<0.0001) (Table 3 & 4).

**Table 3:** Distribution of haplotypes frequencies for *TNF*-α promoter polymorphisms among generalized vitiligo patients and controls.

Haplotype (−238G/A,−308 G/A, −857 C/T,−863 C/A and −1031 T/C)	Generalized VitiligoPatients (Freq. %)(n = 1454)	Controls (Freq. %)(n = 1936)	*p* for Association	*p*_(global)_	Odds ratio(95% CI)
A A C A T	49.92 (0.034)	13.88 (0.007)	2.33e-012	<0.0001	6.547 (3.593∼11.929)
A A C C T	94.79 (0.065)	21.40 (0.011)	5.67e-025		8.420 (5.232∼13.551)
A A T C C	60.38 (0.042)	2.04 (0.001)	1.48e-023		54.818 (13.554∼221.703)
A A T C T	71.17 (0.049)	13.93 (0.007)	4.31e-020		9.511 (5.326∼16.982)
A G C C T	60.72 (0.042)	68.55 (0.035)	0.010890		1.580 (1.108∼2.251)
G A C C T	31.38 (0.022)	83.64 (0.043)	0.036942		0.643 (0.423∼0.977)
G G C A T	83.30 (0.057)	181.62 (0.094)	0.075190		0.782 (0.596∼1.026)
G G C C C	72.25 (0.050)	206.78 (0.107)	0.000113		0.579 (0.438∼0.766)
G G C C T	218.00 (0.150)	778.39 (0.402)	7.54e-033		0.347 (0.291∼0.414)
G G T A T	65.08 (0.045)	51.56 (0.027)	9.25e-006		2.284 (1.571∼3.320)
G G T C C	56.39 (0.039)	81.71 (0.042)	0.271883		1.215 (0.858∼1.722)
G G T C T	144.86 (0.100)	254.60 (0.132)	0.934445		0.991 (0.795∼1.235)

CI represents Confidence Interval,

(Frequency <0.03 in both control & case has been dropped and was ignored in analysis).

**Table 4:** Distribution of haplotypes frequencies for *TNF*-α promoter polymorphisms among localized vitiligo patients and controls.

Haplotype (−238G/A,−308 G/A, −857 C/T,−863 C/A and −1031 T/C)	Localized VitiligoPatients (Freq. %)(n = 482)	Controls (Freq. %) (n = 1936)	*p* for Association	*p*_(global)_	Odds ratio (95% CI)
A A C A T	22.81 (0.047)	13.88 (0.007)	9.51e-012	<0.0001	7.520 [3.824∼14.787]
A A C C T	40.56 (0.084)	21.40 (0.011)	9.52e-022		9.047 [5.296∼15.453]
A A T C C	37.87 (0.079)	2.04 (0.001)	6.66e-016		88.928 [21.647∼365.322]
A A T C T	33.24 (0.069)	13.93 (0.007)	2.89e-020		11.220 [5.944∼21.180]
A G C C C	20.19 (0.042)	21.79 (0.011)	7.93e-007		4.193 [2.267∼7.758]
A G C C T	17.72 (0.037)	68.55 (0.035)	0.648433		1.132 [0.664∼1.930]
G A C C T	5.60 (0.012)	83.64 (0.043)	0.002174		0.282 [0.119∼0.668]
G G C A C	20.16 (0.042)	20.87 (0.011)	4.39e-007		4.373 [2.348∼8.144]
G G C A T	21.90 (0.045)	181.62 (0.094)	0.002400		0.499 [0.316∼0.788]
G G C C C	19.17 (0.040)	206.78 (0.107)	3.43e-005		0.375 [0.232∼0.606]
G G C C T	74.89 (0.155)	778.39(0.402)	3.35e-021		0.291 [0.223∼0.380]
G G T A T	15.29 (0.032)	51.56 (0.027)	0.369915		1.304 [0.729∼2.330]
G G T C C	17.54 (0.036)	81.71(0.042)	0.795328		0.933 [0.550∼1.580]
G G T C T	51.54 (0.107)	254.60(0.132)	0.375415		0.865 [0.628∼1.192]

CI represents Confidence Interval,

(Frequency <0.03 in both control & case has been dropped and was ignored in analysis).

The susceptible haplotypes: AACAT, AACCT, AATCC, AATCT and AGCCT were more frequently observed in generalized vitiligo patients as compared to controls and were found to increase the risk of generalized vitiligo as suggested by odds ratio (Table 3). However, the non-susceptible haplotypes: GGCAT, GGCCC, GGCCT, GGTCC, GGTCT were more frequently observed in controls as compared to generalized vitiligo patients (Table 3).

Furthermore, susceptible haplotypes: AACAT, AACCT, AATCC, AATCT, AGCCC and AGCCT were more frequently observed in localized vitiligo patients as compared to controls and were found to increase the risk of localized vitiligo as suggested by odds ratio (Table 4); however, the non-susceptible haplotypes: GGCAT, GGCCC, GGCCT, GGTAT and GGTCC were more frequently observed in controls as compared to localized vitiligo patients (Table 4).

### Age of Onset of Vitiligo and *TNF*-α Promoter Haplotypes in Patients with Vitiligo

**Figure 1:**
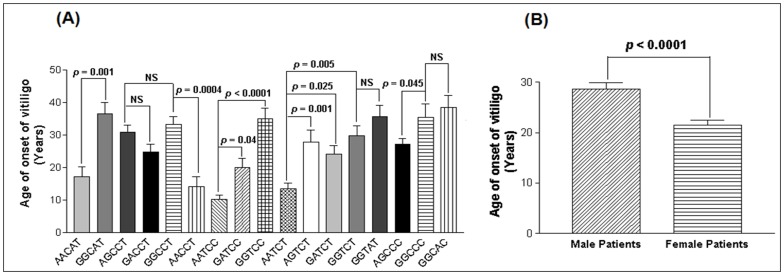
Age of onset of the disease in vitiligo patients. (**A**) Comparison of age of onset of the disease (years) with respect to *TNF*-α promoter haplotypes in 977 vitiligo patients. (**B**) Comparison of age of onset of the disease (years) with respect to gender differences in 451 male patients and 526 female patients with vitiligo.

When age of onset of the disease was correlated with the *TNF*-α promoter haplotypes, patients with AACAT, AACCT, AATCC and AATCT haplotypes showed early onset of the disease as compared to GGCAT, GGCCT, GGTCC and GGTCT (*p* = 0.001, *p* = 0.0004, *p*<0.0001 and *p* = 0.005 respectively) (Figure 1A). Patients with haplotype AATCC had an early onset of the disease as compared to GATCC haplotype (*p* = 0.04). Moreover, patients with haplotype AATCT showed early onset of the disease as compared to AGTCT and GATCT haplotypes (*p* = 0.001 and *p* = 0.025 respectively) (Figure 1A). Also, patients with AGCCC haplotype had an early onset of the disease as compared to GGCCC haplotype (*p* = 0.045); however, there was no significant difference in age of onset of the disease for haplotype AGCCT as compared to GACCT and GGCCT haplotypes (*p* = 0.147 and *p* = 0.481 respectively) (Figure 1A). Patients with haplotypes GGTCT and GGCCC showed no significant difference in age of onset of the disease as compared to GGTAT and GGCAC haplotypes (*p* = 0.248 and *p* = 0.582 respectively) (Figure 1A). Interestingly, when male and female vitiligo patients were analyzed for age of onset of the disease, female patients had significant early onset of the disease as compared to the male patients (*p*<0.0001) (Figure 1B).

### Relative Gene Expression of *TNF*-α in Patients with Vitiligo and Controls

**Figure 2:**
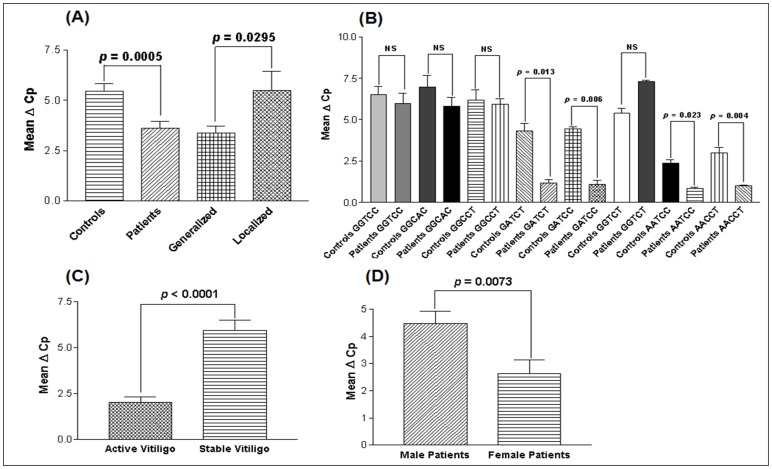
Relative gene expression of *TNF*-α in controls and vitiligo patients. (**A**) Expression of *TNF*-α transcripts in 174 controls, 157 vitiligo patients, 115 generalized vitiligo patients and 42 localized vitiligo patients, as suggested by Mean ΔCp. (**B**) Expression of *TNF*-α transcripts with respect to *TNF*-α promoter haplotypes in 157 vitiligo patients and 174 controls, as suggested by Mean ΔCp. (**C**) Expression of *TNF*-α transcripts with respect to activity of the disease in 108 patients with active vitiligo and 49 patients with stable vitiligo, as suggested by Mean ΔCp. (**D**) Expression of *TNF*-α transcripts with respect to gender differences in 68 male patients and 89 female patients with vitiligo, as suggested by Mean ΔCp.

Comparison of the findings showed significantly increased expression of *TNF*-α transcripts in 157 vitiligo patients than in 174 unaffected controls after normalization with *GAPDH* expression as suggested by mean ΔCp values (*p* = 0.0005) (Figure 2A). Moreover, generalized vitiligo patients showed significant higher expression of *TNF*-α transcripts as compared to localized vitiligo patients (*p* = 0.0295) (Figure 2A). The 2**^−^** ^ΔΔCp^ analysis showed approximately 0.445 fold change in the expression of *TNF*-α transcript in patients as compared to controls (Figure S3).

### Correlation of *TNF-α* Promoter Genotypes and Haplotypes with its Transcript Levels

**Figure 3:**
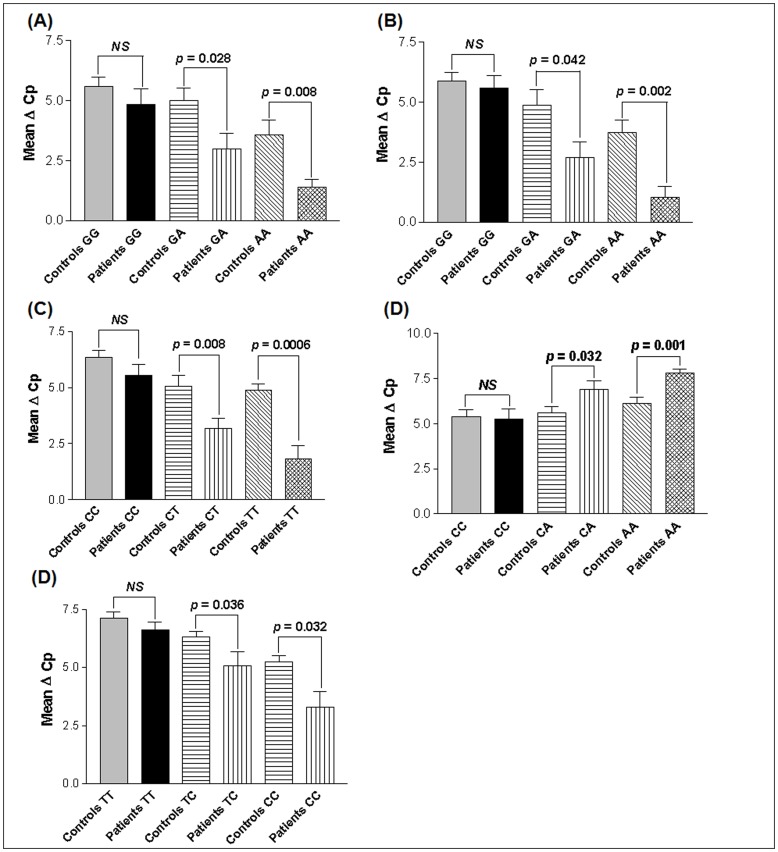
Relative gene expression of *TNF*-α with respect to promoter genotypes in controls and vitiligo patients. (**A**) Expression of *TNF*-α transcripts with respect to −238 G/A genotypes in 157 vitiligo patients and 174 controls, as suggested by Mean ΔCp. (**B**) Expression of *TNF*-α transcripts with respect to −308 G/A genotypes in 157 vitiligo patients and 174 controls, as suggested by Mean ΔCp. (**C**) Expression of *TNF*-α transcripts with respect to −857 C/T genotypes in 157 vitiligo patients and 174 controls, as suggested by Mean ΔCp. (**D**) Expression of *TNF*-α transcripts with respect to −863 C/A genotypes in 157 vitiligo patients and 174 controls, as suggested by Mean ΔCp. (**E**) Expression of *TNF*-α transcripts with respect to −1031 T/C genotypes in 157 vitiligo patients and 174 controls, as suggested by Mean ΔCp.

The expression levels of *TNF-α* for the −238 GG genotype did not differ significantly between vitiligo patients and controls (*p* = 0.294). However, patients with −238 GA and AA genotypes revealed higher *TNF-α* transcript levels as compared to controls (*p* = 0.028 and *p* = 0.008 respectively) (Figure 3A). Also, *TNF-α* expression differed significantly between patients and controls for −308 GA and AA genotypes (*p* = 0.042 and *p* = 0.002 respectively) whereas for GG genotype the expression did not differ (*p* = 0.064) (Figure 3B). For −857 C/T SNP, the *TNF-α* expression was higher in patients with CT and TT genotypes (*p* = 0.008 and *p* = 0.001 respectively) whereas the expression did not differ with CC genotypes (*p* = 0.173) (Figure 3C). However, *TNF-α* expression was reduced in patients with −863 CA and AA genotypes (*p* = 0.032 and *p* = 0.001 respectively) whereas the expression did not differ with CC genotypes (*p* = 0.828) (Figure 3D). For −1031 T/C SNP, the *TNF-α* expression was higher in patients with TC and CC genotypes (*p* = 0.036 and *p* = 0.032 respectively) whereas the expression did not differ with TT genotypes (*p* = 0.284) (Figure 3E).

Further, the expression levels of *TNF*-α were analyzed with respect to haplotypes generated from the five investigated promoter polymorphisms of *TNF*-α (Figure 2B). Interestingly, *TNF*-α expression was significantly increased for the haplotypes: GATCT, GATCC, AATCC and AACCT in vitiligo patients as compared to controls (*p* = 0.013, *p* = 0.006, *p* = 0.023 and *p* = 0.004 respectively); however, no significant difference was observed in *TNF*-α expression for the haplotypes: GGTCC, GGCAC, GGCCT and GGTCT (*p* = 0.517, *p* = 0.258, *p* = 0.790 and *p* = 0.456 respectively).

In addition, we analyzed the *TNF*-α expression based on the progression of the disease i.e. active vitiligo and stable vitiligo (Figure 2C). Active vitiligo patients showed significantly increased expression of *TNF*-α transcripts as compared to the patients with stable vitiligo (*p*<0.0001). To check the susceptibility of the disease based on the gender differences *TNF*-α expression was analyzed for male and female vitiligo patients. Female patients with vitiligo showed significantly higher *TNF*-α expression as compared to male patients (*p* = 0.0073) (Figure 2D).

### Functional Correlation of *TNF*-α Promoter Polymorphisms with its Levels in the Serum

**Figure 4:**
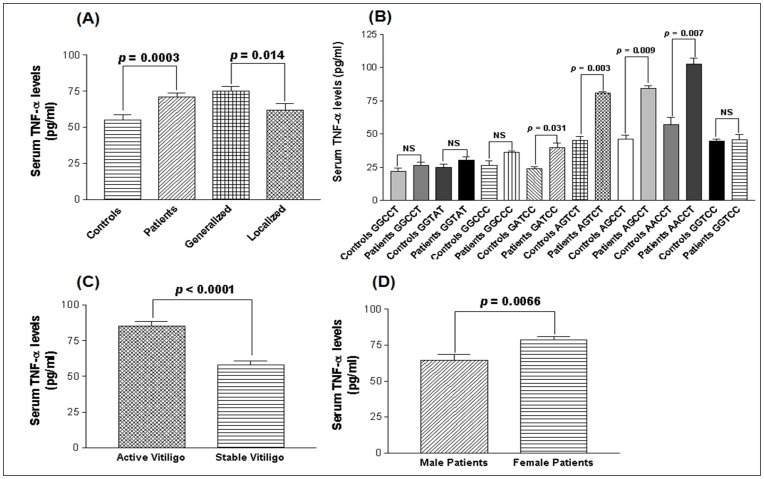
Serum TNF-α levels in controls and vitiligo patients. (**A**) Comparison of sTNF-α levels (pg/ml) in 236 controls, 214 vitiligo patients, 158 generalized vitiligo patients and 56 localized vitiligo patients, as determined by ELISA. (**B**)**.** Comparison of sTNF-α levels (pg/ml) with respect to *TNF*-α promoter haplotypes in 214 vitiligo patients and 236 controls, as determined by ELISA. (**C**) Comparison of sTNF-α levels (pg/ml) with respect to activity of the disease in 150 patients with active vitiligo and 64 patients with stable vitiligo, as determined by ELISA. (**D**) Comparison of sTNF-α levels (pg/ml) with respect to gender differences in 97 male patients and 117 female patients with vitiligo, as determined by ELISA.

To find any functional correlation of the investigated *TNF*-α promoter polymorphisms with its level in the serum, TNF-α levels were measured in 214 vitiligo patients and 236 unaffected controls. Vitiligo patients showed significant increased serum TNF-α (sTNF-α) levels as compared to controls (*p* = 0.0003) (Figure 4A). Moreover, when the patient subgroups were analyzed with respect to sTNF-α levels, patients with generalized vitiligo had significantly higher sTNF-α levels as compared to localized vitiligo (*p* = 0.014) (Figure 4A).

**Figure 5:**
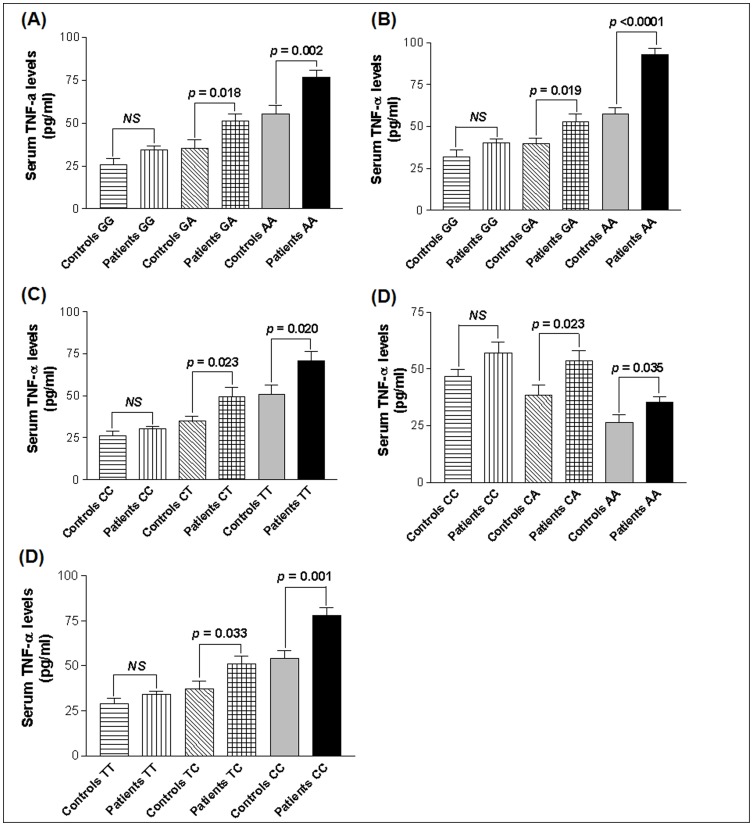
Serum TNF-α levels with respect to promoter genotypes in controls and vitiligo patients. (**A**) Comparison of sTNF-α levels (pg/ml) with respect to −238 G/A genotypes in 214 vitiligo patients and 236 controls, as determined by ELISA. (**B**) Comparison of sTNF-α levels (pg/ml) with respect to −308 G/A genotypes in 214 vitiligo patients and 236 controls, as determined by ELISA. (**C**) Comparison of sTNF-α levels (pg/ml) with respect to −857 C/T genotypes in 214 vitiligo patients and 236 controls, as determined by ELISA. (**D**) Comparison of sTNF-α levels (pg/ml) with respect to −863 C/A genotypes in 214 vitiligo patients and 236 controls, as determined by ELISA. (**E**) Comparison of sTNF-α levels (pg/ml) with respect to −1031 T/C genotypes in 214 vitiligo patients and 236 controls, as determined by ELISA.

For the −238 G/A SNP, the GA and AA genotypes showed significant increase in sTNF-α levels in vitiligo patients as compared to controls (*p* = 0.018 and 0.002 respectively). However, for the −238 GG genotype sTNF-α levels did not differ between these groups (*p* = 0.062) (Figure 5A). Further, the −308 GA and AA genotypes showed significant increase in sTNF-α levels in patients (*p* = 0.019 and *p*<0.0001 respectively) whereas the levels were not differed for GG genotypes between patients and controls (*p* = 0.082) (Figure 5B). For −857 C/T SNP, the sTNF-α levels were high in patients with CT and TT genotypes (*p* = 0.023 and *p* = 0.020 respectively) whereas the levels did not differ with CC genotypes (*p* = 0.246) (Figure 5C). However, sTNF-α levels were reduced in patients with −863 CA and AA genotypes (*p* = 0.023 and *p* = 0.035 respectively) whereas the levels did not differ with CC genotypes (*p* = 0.067) (Figure 5D). For −1031 T/C SNP, the sTNF-α levels were high in patients with TC and CC genotypes (*p* = 0.033 and *p* = 0.001 respectively) whereas the levels did not differ with TT genotypes (*p* = 0.211) (Figure 5E).

In vitiligo patients, the *TNF*-α haplotypes: GATCC, AGTCT, AGCCT and AACCT were found to increase sTNF-α levels (*p* = 0.031, *p* = 0.003, *p* = 0.009 and *p* = 0.007 respectively) with susceptible alleles (−238A, −308A, −857T and −1031C) as compared to controls (Figure 4B). However, no significant difference was observed in sTNF-α levels for the haplotypes: GGCCT, GGTAT, GGCCC, and GGTCC (*p* = 0.217, *p* = 0.150, *p* = 0.153 and *p* = 0.868 respectively) (Figure 4B).

Furthermore, when haplotypes were analyzed for sTNF-α levels in the patients based on the disease activity, patients with active vitiligo showed significantly higher sTNF-α levels as compared to stable vitiligo (*p*<0.0001) (Figure 4C). Additionally, when the male and female patients were analyzed with respect to sTNF-α levels, female patients had significantly higher levels of sTNF-α as compared to male patients (*p* = 0.0066) (Figure 4D).

## Discussion

Vitiligo susceptibility is a complex genetic trait that may include genes involved in melanin biosynthesis, response to oxidative stress and regulation of autoimmunity. The importance of genetic factors for vitiligo susceptibility is evident by reports of its significant familial association [18],[19]. Our previous study suggests that 22% of Gujarat vitiligo patients exhibit positive family history and 14% patients have at least one first-degree relative affected [20]. Autoimmunity has been suggested to play a major role in the pathogenesis of vitiligo. Destruction of melanocytes due to an autoimmune response in vitiligo can be either through cellular and/or humoral immune response [6],[7]. We have also shown that 66% of vitiligo patients possessed anti-melanocyte antibodies in their circulation as compared to control population [21]. Recently, we have shown positive association of HLA-A*33:01, HLA-B*44:03, and HLA-DRB1*07:01 with vitiligo patients from North India and Gujarat suggesting an autoimmune link of vitiligo in these cohorts [22]. The genotype-phenotype correlation of *CTLA*-4 and *IL*-4 gene polymorphisms also supported the autoimmune pathogenesis of vitiligo in Gujarat population [23],[24], whereas our earlier studies on *MBL*-2, *ACE, PTPN*22 polymorphisms did not show significant association [25]–[27].

Cytokines are important mediators of immunity and there is now convincing evidence that cytokines also have an important role in the pathogenesis of autoimmunity [28]. The cytokines mRNA and protein levels depend on both genetic and environmental factors. Analysis of cytokine gene polymorphisms would be able to detect genetic abnormality of cytokine regulation and hence establishment of genotype-phenotype correlation may be important in unraveling the disease pathogenesis. The promoter polymorphisms of *TNF*-α are reported to be involved in modulating expression of *TNF*-α gene which may be responsible for melanocyte death.

TNF-α, is an important multifunctional cytokine secreted by macrophages, T-lymphocytes, fibroblasts and keratinocytes with wide-ranging biological effects of protection from infection, surveillance against tumors and stimulation of inflammatory responses. In the epidermis, the epidermal melanin unit consists of the close interaction of a melanocyte and an associated pool of keratinocytes. Close relationship between these two cell types is important for melanocyte survival and differentiation mainly as keratinocyte-derived cytokines act on melanocytes via specific receptors [29]. Keratinocytes synthesize cytokines, such as TNF-α, IL-1a, IL-6, and transforming growth factor-b (TGF-b), which are paracrine inhibitors of human melanocyte proliferation and melanogenesis [29]. However, primary role of TNF-α is in the regulation of immune cells and its overproduction has been implicated in a variety of human diseases including autoimmune disorders and cancer [30]. *In vitro*, direct analysis of skin T cells from margins of vitiliginous skin show that polarized type-1 T cells (CD4+ and particularly CD8+), which predominantly secrete interferon (IFN)-γ and TNF-α are associated with the destruction of melanocytes during active vitiligo [31]. In vitiligo affected skin, a significantly higher expression of TNF-α [32],[29], IL-6 [29], IFN-γ [32] was detected compared with healthy controls and perilesional, non-lesional skin [29] indicating that cytokine imbalance plays an important role in the depigmentation process of vitiligo.

It has been reported that cytokines such as IFN-γ and TNF-α can initiate apoptosis and thus lead to melanocyte death in the context of autoimmunity [33]. In addition, IFN-γ and TNF-α induce the expression of intercellular adhesion molecule-1 (ICAM-1) on the cell-surface of melanocytes [34]. The increased expression of ICAM-1 on the melanocytes enhances T cell/melanocyte attachment in the skin and thus may result in destruction of melanocytes in vitiligo [35],[36]. TNF-α also has the capacity to inhibit melanogenesis through an inhibitory effect on tyrosinase and tyrosinase related proteins [37].

Thus, it becomes pertinent to study all *TNF*-α promoter polymorphisms in adequate number of vitiligo patients and controls to elucidate the role of these polymorphisms in vitiligo susceptibility and to analyze the possible genotype - phenotype correlation. Here, we report that *TNF-α* −238, −308, −857, −863 and −1031 promoter polymorphisms are significantly associated with Gujarat vitiligo patients. Our results clearly suggest the important role of TNF-α in pathogenesis of vitiligo. Vitiligo patients showed significant increase in *TNF*-α transcript and protein levels as compared to controls suggesting that melanocyte death in patients could be triggered due to the increased TNF-α levels.

For the first time we report that generalized vitiligo has significantly higher *TNF*-α transcript and protein levels as compared to localized vitiligo patients which indicate involvement of autoimmunity in precipitation of generalized vitiligo. Our results also indicate that active vitiligo patients have significantly higher *TNF*-α transcript and protein levels as compared to the patients with stable vitiligo which signifies the role of TNF-α in disease progression. Our results also suggest that there are significantly higher transcript and protein levels of TNF-α in female patients as compared to male patients. Moreover, female patients have an early onset as compared to male patients suggestive of the fact that females have increased susceptibility towards vitiligo as compared to males, implicating gender biasness in the development of autoimmunity [38]–[40].

The *TNF*-α −308 G/A and −238 G/A polymorphisms were found to influence serum TNF-α levels in patients with sarcoidosis of Asian Indian population [41] and our results are in line with this study. Interestingly, we found that the five promoter polymorphisms influence *TNF*-α expression wherein *TNF*-α −238, −308, −857 and −1031 were found to increase whereas −863 was found to decrease the expression. Furthermore, a genotype-phenotype study carried out on SLE patients showed increased *TNF*-α transcript levels with −238 AA and GA genotypes as compared to GG genotypes [42]. In particular, in the present study when combined effect of various genotypes was analyzed in the form of haplotypes, AATCC haplotype was found to be the highest risk combination observed for the disease. Intrestingly, it has all susceptible alleles except −863A which is reported to decrease the levels of TNF-α. The −863 C/A polymorphism was associated with serum TNF-α levels, carriers of the rare ‘A’ allele having a significantly lower TNF-α levels in Swedish population [43]. The −863A allele was associated with 31% lower transcriptional activity in chloramphanicol acetyltransferase (CAT) reporter gene studies in human hepatoblastoma (HepG2) cells [43]. Moreover, the haplotype analysis revealed the degree of susceptibility to the disease as predicted by the odds ratio with generalized vitiligo: AATCC >AATCT >AACCT >AACAT >AGCCT and AATCC >AATCT >AACCT >AACAT >AGCCC for localized vitiligo. Also, the age of onset analysis of the disease suggested the haplotypes involved in the early age of onset in patients with vitiligo are those involved in high degree of susceptibility of the disease: AATCC >AATCT >AACCT >AACAT >AGCCC >AGCCT.

LD analysis suggests that *TNF-α* −238 G/A & −308 G/A polymorphisms in moderate LD association as compared to the other investigated polymorphisms and are strongly associated with the disease risk in patients as suggested by the odds ratio. Moreover, the haplotype analysis showed the presence of haplotypes involving the susceptible alleles of *TNF-α* −238 and −308 polymorphisms, having increased levels of TNF-α in patients as compared to controls.

The region between −254 to −230 contains a regulatory sequence that acts as a *TNF-*α repressor site [44]. Previously, Bayley et al. [45] showed that −238A allele increases the *TNF*-α expression in transfected B cell line Raji and monocytic cell line U937 with a series of mutant constructs within the repressor region, including one where the –238G allele was replaced by a 10 bp linker sequence containing the –238A mutant allele. U937 and Raji cells containing the –238 minor A allele construct showed consistent increase (1.4 to 1.8-fold) in both basal and inducible promoter activity suggesting that the –238 SNP and the region surrounding it could be important in *TNF*-α regulation and a mutation at position –238 could disrupt its regulation [45].

Kroeger et al. [46] first showed that −308A allelic form gave a two-fold higher level of transcription than the −308G form in PMA-stimulated Jurkat and U937 cells using a luciferase reporter gene assay suggesting that the −308 G/A polymorphism plays an important role in the altered *TNF*-α gene expression. Furthermore, Wilson et al. [47] also showed functional significance of −308G/A polymorphism by investigating its effects on *TNF*-α transcription using reporter gene assays suggesting that the −308 minor A allele is a much more powerful transcriptional activator than the common allele in a human B cell line.

The study of *TNF*-α −308 G/A polymorphism in Iranian population have revealed significant association of −308A allele with vitiligo patients [48] and these results are in line with our study however, a previous study of Turkish population suggested that *TNF-α* −308 G/A polymorphism has no significant influence on vitiligo susceptibility [49]. These contradictory reports may be because of the differences in ethnicity of the studied populations. However, both the studies involved less sample size and hence the association results needed further confirmation. Furthermore, there are no reports available on the effect of these *TNF*-α promoter polymorphisms on its expression in vitiligo patients and the present study revealed the significant role of these promoter polymorphisms on the levels of TNF-α which might be playing a central role in vitiligo pathogenesis.

It has been known that the ROS microenvironment decides the fate of a cell for TNF-α mediated apoptosis [50]. Our earlier reports with other studies suggest that the high oxidative environment prevails in vitiligo patients for the melanocyte destruction [51–53]. The destruction of melanocytes might be due to the increased secretion of TNF-α which further increases ROS and thus may lead to an early/defective apoptosis of the melanocytes via TNF-α mediated pathway. The possiblity of the TNF-α secretion is very high since the keratinocytes (a source of TNF-α) surround these melanocytes forming a melanin epidermal unit and thus affect its proliferation and melanogenesis process.

Disturbances in TNF-α metabolism have been well documented and found to be associated with several other autoimmune and infectious diseases such as rheumatoid arthritis [54], systemic lupus erythematosus [55], crohn’s disease [56], cerebral malaria [57] and lesihmaniasis [58]. Previously North Indian and Caucasian studies revealed strong association of −308 G/A polymorphism with T1DM [59],[60]. A study with psoriatic arthritis patients in Caucasian population for the five promoter polymorphisms suggested significant association of −238 G/A polymorphism with patients being −238 (A) variant, a significant risk factor for the disease [61]. The *TNF*-α −308 G/A polymorphism was significantly associated with susceptibility to asthma in patients of South Iran and with susceptibility to inflammatory bowel disease in European population [62],[63]. A metaanalysis study suggested that *TNF*-α −238G/A and −308G/A polymorphisms might be used as biomarkers for psoriasis risk prediction [64]. Furthermore, a study involving 22 SNPs in Caucasian patients with Graves’ disease (GD) showed significant association of *TNF*-α −238G/A and −308G/A polymorphisms [65].

Simon and Burgor-Vargas [66] described a patient with ankylosing spondylitis (AS) and vitiligo who was treated with infliximab (a chimeric monoclonal anti-TNF antibody), which resulted in gradual fading of vitiligo lesions suggesting that *TNF-α* was involved in the pathogenesis of vitiligo. Alghamdi et al. [13] also showed the effect of anti TNF-α agents: infliximab, etanercept, and adalimumab in generalized vitiligo patients. The patients did not develop any new depigmented patches during treatment or at the six-month follow-up. These reports signify the involvement of TNF-α in vitiligo pathogenesis.

In conclusion, our findings suggest that the increased TNF-α levels in vitiligo patients could result, at least in part, from variations at the genetic level. For the first time, we show that the promoter polymorphisms of the *TNF*-α gene influence the expression both at transcriptional as well as translational levels in vitiligo. The study also emphasizes the influence of TNF-α on the disease progression, onset of the disease and gender biasness for developing vitiligo. More detailed studies regarding role of TNF-α in precipitation of vitiligo and the development of effective anti-TNF-α agents may prove to be useful as preventive/ameliorative therapies.

## Supporting Information

Figure S1 **PCR-RFLP analysis of** ***TNF*** **-α promoter polymorphisms. (A)** PCR-RFLP analysis of *TNF*-α −238 G/A polymorphism on 3.5% agarose gel: lanes: 1, 2, 3, 4, 5, 11, 12, 13, 14 & 18 show homozygous (GG) genotypes; lanes: 16, 17 & 19 show heterozygous (GA) genotypes; lanes: 6, 7, 8, 9 10 & 15 show homozygous (AA) genotypes; lane M shows 100 bp DNA ladder. **(B)** PCR-RFLP analysis of *TNF*-α −308 G/A polymorphism on 10% polyacrylamide gel: lanes: 1, 2, 4 & 5 show heterozygous (GA) genotypes; lanes: 3 & 6 show homozygous (GG) genotypes; lane M shows 100 bp DNA ladder. **(C)** PCR-RFLP analysis of *TNF*-α −857 C/T polymorphism on 10% polyacrylamide gel: lanes: 1, 2, 4, 5 & 6 show heterozygous (CT) genotypes; lane: 3 shows homozygous (GG) genotype; lane M shows 100 bp DNA ladder. **(D)** PCR-RFLP analysis of *TNF*-α −863 C/A polymorphism on 10% polyacrylamide gel: lanes: 1, 3, 4, 5 & 6 show homozygous (CC) genotypes; lane: 2 shows heterozygous (CA) genotype; lane M shows 100 bp DNA ladder. **(E)** PCR-RFLP analysis of *TNF*-α −1031 T/C polymorphism on 2.0% agarose gel: lanes: 4 & 5 show homozygous (TT) genotypes; lanes: 6 & 7 show heterozygous (TC) genotypes; lanes: 1, 2 & 3 show homozygous (CC) genotype; lane M shows 100 bp DNA ladder.
(TIF)

Figure S2 **Linkage disequilibrium analysis of** ***TNF*** **-α promoter polymorphisms. (A)** Linkage disequilibrium (D’) among *TNF*-α promoter SNPs in generalized vitiligo patients and controls from Gujarat population. **(B)** Linkage disequilibrium (D’) among *TNF*-α promoter SNPs in localized vitiligo patients and controls from Gujarat population.
(TIF)

Figure S3 **Expression fold change of** ***TNF*** **-α transcript in 157 vitiligo patients against 174 controls showed 0.445 fold change as determined by 2^−^** ^ΔΔ**Cp**^ **method.**
(TIF)

Table S1 **Demographic characteristics of vitiligo patients and unaffected controls of Gujarat.**
(DOC)

Table S2 **Primers used for** ***TNF*** **-α promoter SNPs genotyping and gene expression analysis.**
(DOC)

Table S3 **Pairwise linkage disequilibrium (D’) values between** ***TNF*** **-α SNPs with >3% minor allele frequencies within generalized vitiligo patients and controls from Gujarat population.**
(DOC)

Table S4 **Pairwise linkage disequilibrium (D’) values between** ***TNF*** **-α SNPs with >3% minor allele frequencies within localized vitiligo patients and controls from Gujarat population.**
(DOC)

## References

[1] Taieb A, Picardo M (2009) Vitiligo. N. Engl. J. Med. 360: 160–169. 10.1056/NEJMcp0804388 19129529

[2] Spritz RA (2008) The genetics of generalized vitiligo. Curr. Dir. Autoimmun. 10: 244–257. 10.1159/000131501 18460890

[3] Das SK, Majumder PP, Chakraborty R, Majumdar TK, Haldar B (1985) Studies on vitiligo: Epidemiological profile in Calcutta, India. Genet. Epidemiol. 2: 71–78. 10.1002/gepi.1370020107 4054593

[4] Alkhateeb A, Fain PR, Thody A, Bennett DC, Spritz RA (2003) Epidemiology of vitiligo and associated autoimmune diseases in Caucasian probands and their relatives. Pigment Cell Res. 16: 208–214. 10.1034/j.1600-0749.2003.00032.x 12753387

[5] Laberge G, Mailloux CM, Gowan K, Holland P, Bennett DC, et al (2005) Early disease onset and increased risk of other autoimmune diseases in familial generalized vitiligo. Pigment Cell Res. 18: 300–305. 10.1111/j.1600-0749.2005.00242.x 16029422

[6] Shajil EM, Chatterjee S, Agrawal D, Bagchi T, Begum R (2006a) Vitiligo: Pathomechanisms and genetic polymorphism of susceptible genes. Ind. J. Exp. Biol. 44: 526–539. 16872041

[7] Kemp EH, Waterman EA, Weetman AP (2001) Immunological pathomechanisms in vitiligo. Expert. Rev. Mol. Med. 23: 1–22. 10.1017/S1462399401003362 14585144

[8] Rosenberg SA (1997) Cancer vaccines based on the identification of genes encoding cancer regression antigens. Immunol. Today. 18: 175–182. 10.1016/s0167-5699(97)84664-6 9136454

[9] Spritz RA (2011) Recent progress in the genetics of generalized vitiligo. J Genet and Genom. 38: 271–278. 10.1016/j.jgg.2011.05.005 PMC3513342 21777851

[10] Spritz RA (2007) The genetics of generalized vitiligo and associated autoimmune diseases. Pigment Cell Res. 20: 271–278. 10.1111/j.1600-0749.2007.00384.x 17630960

[11] Spritz RA (2010) The genetics of generalized vitiligo: autoimmune pathways and an inverse relationship with malignant melanoma. Genome Medicine. 2: 78. 10.1186/gm199 PMC2988443 20959028

[12] Gupta S, Gollapudi S (2006) Molecular mechanisms of TNF-a-induced apoptosis in 136 naive and memory T cell subsets. Autoimmun Rev. 5: 264–268. 10.1016/j.autrev.2005.09.007 16697967

[13] Alghamdi KM, Khurrum H, Taieb A, Ezzedine K (2012) Treatment of generalized vitiligo with anti-TNF-α Agents. J Drugs Dermatol. 11: 534–539. 22453596

[14] Spriggs DR, Deutsch S, Kufe DW (1992) Genomic structure, induction, and production of TNF-alpha. Immunol Ser. 56: 3–34. 1550865

[15] Shi YY, He L (2005) SHEsis, a powerful software platform for analyses of linkage disequilibrium, haplotype construction, and genetic association at polymorphism loci. Cell Res. 15: 97–98. 10.1038/sj.cr.7290272 15740637

[16] Barrett JC, Fry B, Maller J, Dally MJ (2005) Haploview: analysis and visualization of LD and haplotype maps. Bioinformatics. 21: 263–265. 10.1093/bioinformatics/bth457 15297300

[17] Faul F, Erdfelder E, Lang AG, Buchner A (2007) G*Power 3: A flexible statistical power analysis program for the social, behavioral, and biomedical sciences. Behav. Res. Methods. 39: 175–191. 10.3758/bf03193146 17695343

[18] Nordlund JJ (1997) The epidemiology and genetics of vitiligo. Clin Dermatol. 15: 875–878. 10.1016/s0738-081x(97)00128-4 9404690

[19] Kim SM, Chung HS, Hann SK (1998) The genetics of vitiligo in Korean patients. Int J Dermatol. 37: 908–910. 10.1046/j.1365-4362.1998.00549.x 9888330

[20] Shajil EM, Agrawal D, Vagadia K, Marfatia YS, Begum R (2006b) Vitiligo: Clinical profiles in Vadodara, Gujarat. Ind. J. Dermatol. 51: 100–104.

[21] Shajil EM (2007) Biochemical basis and genetic association studies of selected single nucleotide polymorphisms in catalase and glutathione peroxidase genes in vitiligo. *Ph.D. thesis*. The M. S. University of Baroda, Vadodara, Gujarat, India.

[22] Singh A, Sharma P, Kar HK, Sharma VK, Tembhre MK, et al (2012) HLA alleles and amino acid signatures of the peptide binding pockets of HLA molecules in Vitiligo. J Invest. Dermatol. 132: 124–134. 10.1038/jid.2011.240 21833019

[23] Dwivedi M, Laddha NC, Imran M, Shah BJ, Begum R (2011) Cytotoxic T-lymphocyte associated antigen-4 (CTLA-4) in isolated vitiligo: a genotype-phenotype correlation. Pigment cell Melanoma Res. 24: 737–740. 10.1111/j.1755-148X.2011.00892.x 21794098

[24] Imran M, Laddha NC, Dwivedi M, Mansuri MS, Singh J, et al (2012) Interleukin-4 genetic variants correlate with its transcript and protein levels in vitiligo patients. Brit J Dermatol. 167: 314–323. 10.1111/j.1365-2133.2012.11000.x 22512783

[25] Dwivedi M, Gupta K, Gulla KC, Laddha NC, Hajela K, et al (2009) Lack of genetic association of promoter and structural variants of Mannan-binding lectin (MBL2) gene with susceptibility to generalized vitiligo. Brit J Dermatol. 161: 63–69. 10.1111/j.1365-2133.2009.09140.x 19416237

[26] Dwivedi M, Laddha NC, Shajil EM, Shah BJ, Begum R (2008) The ACE gene I/D polymorphism is not associated with generalized Vitiligo Susceptibility in Gujarat population. Pigment cell Melanoma Res. 21: 407–408. 10.1111/j.1755-148X.2008.00462.x 18444962

[27] Laddha NC, Dwivedi M, Shajil EM, Prajapati H, Marfatia YS, et al (2008) Association of PTPN22 1858C/T polymorphism with vitiligo susceptibility in Gujarat population. J Dermatol Sci. 49: 260–262. 10.1016/j.jdermsci.2007.10.002 18037273

[28] Feldmann M, Brennan FM, Maini R (1998) Cytokines in autoimmune disorders. Int Rev Immunol. 17: 217–228. 10.3109/08830189809084493 9914949

[29] Moretti S, Spallanzani A, Amato L, Hautmann G, Gallerani I, et al (2002) New insights into the pathogenesis of vitiligo: imbalance of epidermal cytokines at sites of lesions. Pigment Cell Res. 15: 87–92. 10.1034/j.1600-0749.2002.1o049.x 11936274

[30] Locksley RM, Killeen N, Lenardo MJ (2001) The TNF and TNF receptor superfamilies: integrating mammalian biology. Cell. 104: 487–501. 10.1016/s0092-8674(01)00237-9 11239407

[31] Wajkowicz-Kalijska A, van den Wijngaard RM, Tigges BJ, Westerhof W, Ogg GS, et al (2003) Immunopolarization of CD4+ and CD8+ T cells to Type-1-like is associated with melanocyte loss in human vitiligo. Lab Invest. 83: 683–695. 10.1097/01.lab.0000069521.42488.1b 12746478

[32] Grimes PE, Morris R, Avaniss-Aghajani E, Soriano T, Meraz M, et al (2004) Topical tacrolimus therapy for vitiligo: therapeutic responses and skin messenger RNA expression of proinflammatory cytokines. J Am Acad Dermatol. 51: 52–61. 10.1016/j.jaad.2003.12.031 15243524

[33] Huang CL, Nordlund JJ, Boissy R (2002) Vitiligo. A manifestation of apoptosis. Am J Clin Dermatol. 3: 301–308. 10.2165/00128071-200203050-00001 12069635

[34] Yohn JJ, Critelli M, Lyons MB, Norris DA (1990) Modulation of melanocyte intercellular adhesion molecule-1 by immune cytokines. J Invest Dermatol. 90: 233–237. 10.1111/1523-1747.ep12478093 1974278

[35] Al Badri AM (1993) Abnormal expression of MHC class ll and ICAM-l by melanocytes in vitiligo. J Pathol. 169: 203–206. 10.1002/path.1711690205 8095298

[36] Morelli JG, Norris DA (1993) Influence of inflammatory mediators and cytokines on human melanocyte function. J Invest Dermatol. 100: 191–195. 8381839

[37] Martinez-Esparza M, Jimenez-Cervantes C, Solano F, Lozano JA, Garcia-Borron JC (1998) Mechanisms of melanogenesis inhibition by tumor necrosis factor-α in B16–F10 mouse melanoma cells. Eur J Biochem. 255: 139–146. 10.1046/j.1432-1327.1998.2550139.x 9692912

[38] Whitacre CC (2001) Sex differences in autoimmune disease. Nat. Immunol. 2: 777–780. 10.1038/ni0901-777 11526384

[39] Panchanathan R, Choubey D (2012) Murine BAFF expression is up-regulated by estrogen and interferons: Implications for sex bias in the development of autoimmunity. Mol Immunol. 53: 15–23. 10.1016/j.molimm.2012.06.013 PMC3439561 22784990

[40] Afshan G, Afzal N, Qureshi S (2012) CD4+CD25(hi) regulatory T cells in healthy males and females mediate gender difference in the prevalence of autoimmune diseases. Clin Lab. 58 567–571. 22783590

[41] Sharma S, Ghosh B, Sharma SK (2008) Association of TNF polymorphisms with sarcoidosis, its prognosis and tumour necrosis factor (TNF)-alpha levels in Asian Indians. Clin Exp Immunol. 151: 251–259. 10.1111/j.1365-2249.2007.03564.x PMC2276943 18062795

[42] Suárez A, López P, Mozo L, Gutiérrez C (2005) Differential effect of IL10 and TNF{alpha} genotypes on determining susceptibility to discoid and systemic lupus erythematosus. Ann Rheum Dis. 64: 1605–1610. 10.1136/ard.2004.035048 PMC1755257 15800006

[43] Skoog T, van’t Hooft FM, Kallin B, Jovinge S, Boquist S, et al (1999) A common functional polymorphism (C–>A substitution at position −863) in the promoter region of the tumour necrosis factor-alpha (TNF-alpha) gene associated with reduced circulating levels of TNF-alpha. Hum Mol Genet. 8: 1443–1449. 10.1093/hmg/8.8.1443 10400991

[44] Fong CL, Siddiqui AH, Mark DF (1994) Identification and characterization of a novel repressor site in the human tumor necrosis factor alpha gene. Nucleic Acids Res. 22: 1108–1114. 10.1093/nar/22.6.1108 PMC307937 8152914

[45] Bayley JP, de Rooij H, van den Elsen PJ, Huizinga TW, Verweij CL (2001) Functional analysis of linker-scan mutants spanning the −376, −308, −244, and −238 polymorphic sites of the TNFalpha promoter. Cytokine. 14: 316–323. 10.1006/cyto.2001.0902 11497492

[46] Kroeger KM, Carville KS, Abraham LJ (1997) The −308 tumor necrosis factor-alpha promoter polymorphism effects transcription. Mol Immunol. 34: 391–399. 10.1016/s0161-5890(97)00052-7 9293772

[47] Wilson AG, Symons JA, McDowell TL, McDevitt HO, Duff GW (1997) Effects of a polymorphism in the human tumor necrosis factor alpha promoter on transcriptional activation. Proc Natl Acad Sci U S A. 94: 3195–3199. 10.1073/pnas.94.7.3195 PMC20345 9096369

[48] Namian AM, Shahbaz S, Salmanpoor R, Namazi MR, Dehghani F, et al (2009) Association of interferon-gamma and tumor necrosis factor alpha polymorphisms with susceptibility to vitiligo in Iranian patients. Arch Dermatol Res. 301: 21–25. 10.1007/s00403-008-0904-8 18820938

[49] Yazici AC, Erdal ME, Kaya TI, Ikizoglu G, Savasoglu K, et al (2006) Lack of association with TNF-a-308 promoter polymorphism in patients with vitiligo. Arch Dermatol Res. 298: 46–49. 10.1007/s00403-006-0664-2 16691430

[50] Kim JJ, Lee SB, Park JK, Yoo YD (2010) TNF-alpha-induced ROS production triggering apoptosis is directly linked to Romo1 and Bcl-X(L). Cell Death Differ. 17: 1420–1434. 10.1038/cdd.2010.19 20203691

[51] Agrawal D, Shajil EM, Marfatia YS, Begum R (2004) Study on the antioxidant status of vitiligo patients of different age groups in Baroda. Pigment Cell Res. 17: 289–294. 10.1111/j.1600-0749.2004.00149.x 15140075

[52] Shajil EM, Begum R (2006) Antioxidant status of segmental and non segmental vitiligo. Pigment Cell Res. 19: 179–180. 10.1111/j.1600-0749.2006.00299.x 16524434

[53] Schallreuter KU, Wood JM, Berger J (1991) Low catalase levels in the epidermis of patients with vitiligo. J Invest Dermatol. 97: 1081–1085. 10.1111/1523-1747.ep12492612 1748819

[54] Elliot MJ, Mani RN, Feldmann M (1994) Repeated therapy with monoclonal antibody to tumour necrosis factor-α in patients with rheumatoid arthiritis. Lancet. 344: 1125–1127. 10.1016/s0140-6736(94)90632-7 7934495

[55] Jacob CO, Fronek Z, Lewis GD, Koo M, Hansen JA, et al (1990) Heritable major histocomptibility complex class 2 associated difference in production of TNF-α relevance to genetic predisposition to systemic lupus erythematosus. PNAS. 87: 1233–1237. 10.1073/pnas.87.3.1233 PMC53445 2105500

[56] van Dullemenn HM, van Daventer SJ, Hommes DW, Bijl HA, Jansen J, et al (1995) Treatment of Crohn’s disease with anti TNF factor chimeric monoclonal antibody. Gasenterology. 109: 129–135. 10.1016/0016-5085(95)90277-5 7797011

[57] Mc Guire W, Hill AV, Allsopp CE, Greenwood BM, Kwiatkowski D (1994) Variation in TNF-α promoter region associated with susceptibility to central malaria. Nature. 371: 508–510. 10.1038/371508a0 7935762

[58] Carbera M, Shaw MA, Sharples C, Williams H, Castes M, et al (1995) Polymorphism in TNF genes associated with mucocutaneous leishmaniasis. J.Exp.Med. 182: 1259–1264. 10.1084/jem.182.5.1259 PMC2192198 7595196

[59] Kumar R, Goswami R, Agarwal S, Israni N, Singh SK, et al (2007) Association and interaction of the TNF-alpha gene with other pro- and anti-inflammatory cytokine genes and HLA genes in patients with type 1 diabetes from North India. Tissue Antigens. 69: 557–567. 10.1111/j.1399-0039.2007.00817.x 17498265

[60] Noble JA, Valdes AM, Lane JA, Green AE, Erlich HA (2006) Linkage disequilibrium with predisposing DR3 haplotypes accounts for apparent effects of tumor necrosis factor and lymphotoxin-alpha polymorphisms on type 1 diabetes susceptibility. Hum. Immunol. 67: 999–1004. 10.1016/j.humimm.2006.10.002 PMC2481238 17174749

[61] Rahman P, Siannis F, Butt C, Farewell V, Peddle L, et al (2006) TNFalpha polymorphisms and risk of psoriatic arthritis. Ann Rheum Dis. 65: 919–923. 10.1136/ard.2005.039164 PMC1798211 16284098

[62] Kamali-Sarvestani E, Ghayomi MA, Nekoee A (2007) Association of TNF-alpha −308 G/A and IL-4–589 C/T gene promoter polymorphisms with asthma susceptibility in the south of Iran. J Investig Allergol Clin Immunol. 17: 361–366. 18088017

[63] Ferguson LR, Huebner C, Petermann I, Gearry RB, Barclay ML, et al (2008) Single nucleotide polymorphism in the tumor necrosis factor-alpha gene affects inflammatory bowel diseases risk. World J Gastroenterol. 14: 4652–4661. 10.3748/wjg.14.4652 PMC2738789 18698679

[64] Li C, Wang G, Gao Y, Liu L, Gao T (2007) TNF-alpha gene promoter −238G>A and −308G>A polymorphisms alter risk of psoriasis vulgaris: a meta-analysis. J Invest Dermatol. 127: 1886–1892. 10.1038/sj.jid.5700822 17446901

[65] Simmonds MJ, Heward JM, Howson JM, Foxall H, Nithiyananthan R, et al (2004) A systematic approach to the assessment of known TNF-alpha polymorphisms in Graves’ disease. Genes Immun. 5: 267–273. 10.1038/sj.gene.6364066 15057268

[66] Simon JA, Burgos-Vargas R (2008) Vitiligo improvement in a patient with ankylosing spondylitis treated with infliximab. Dermatology. 216: 234–235. 10.1159/000112932 18182816

