# Supplementary figures and images for: Increased Tumor Necrosis Factor (TNF)-α and Its Promoter Polymorphisms Correlate with Disease Progression and Higher Susceptibility towards Vitiligo
Source: PLoS One. 2012 Dec 20;7(12):e52298. doi: 10.1371/journal.pone.0052298 (PMC3527546; doi:10.1371/journal.pone.0052298)

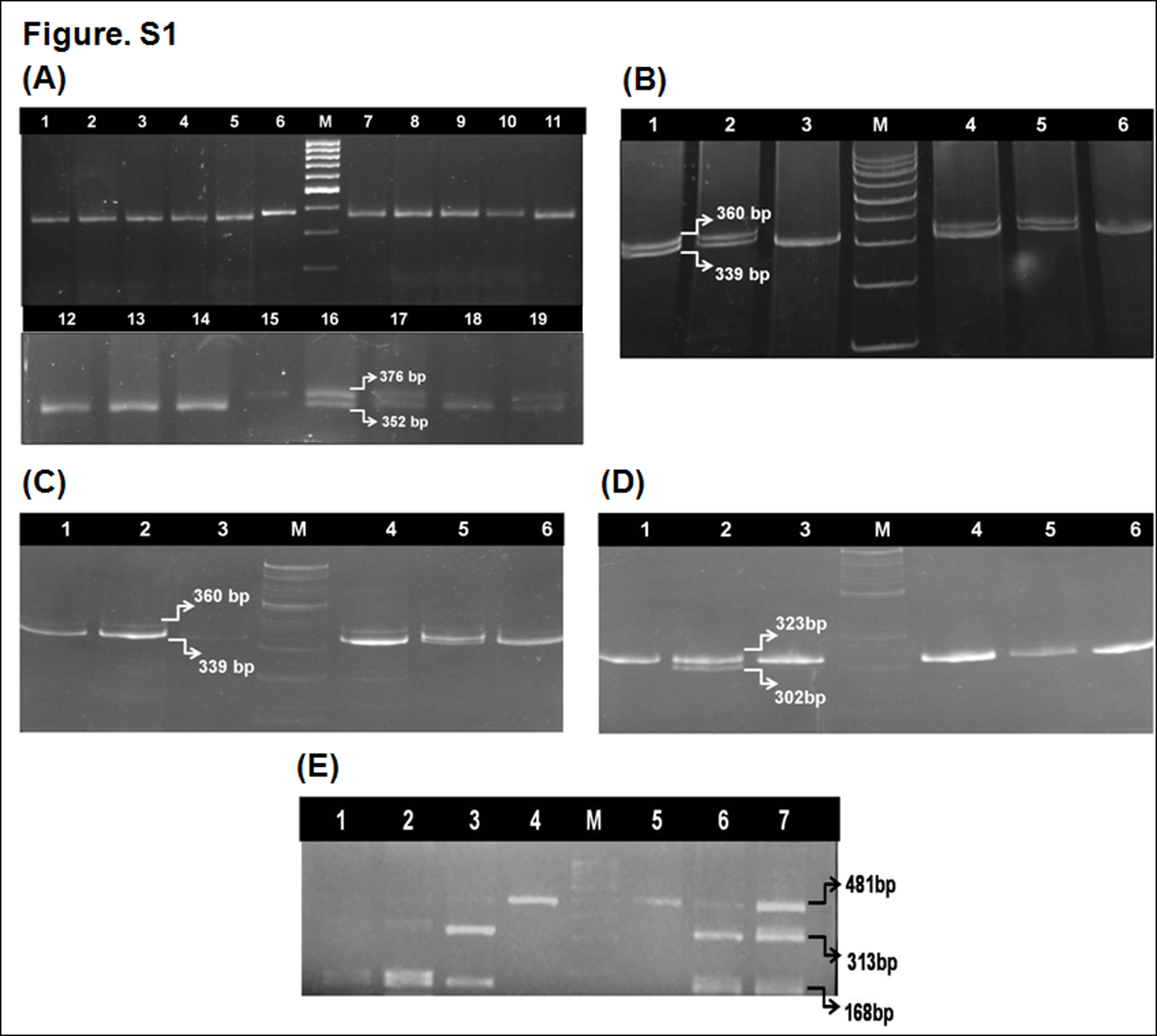

Supplement: Figure S1 — PCR-RFLP analysis of TNF -α promoter polymorphisms. (A) PCR-RFLP analysis of TNF-α −238 G/A polymorphism on 3.5% agarose gel: lanes: 1, 2, 3, 4, 5, 11, 12, 13, 14 & 18 show homozygous (GG) genotypes; lanes: 16, 17 & 19 show heterozygous (GA) genotypes; lanes: 6, 7, 8, 9 10 & 15 show homozygous (AA) genotypes; lane M shows 100 bp DNA ladder. (B) PCR-RFLP analysis of TNF-α −308 G/A polymorphism on 10% polyacrylamide gel: lanes: 1, 2, 4 & 5 show heterozygous (GA) genotypes; lanes: 3 & 6 show homozygous (GG) genotypes; lane M shows 100 bp DNA ladder. (C) PCR-RFLP analysis of TNF-α −857 C/T polymorphism on 10% polyacrylamide gel: lanes: 1, 2, 4, 5 & 6 show heterozygous (CT) genotypes; lane: 3 shows homozygous (GG) genotype; lane M shows 100 bp DNA ladder. (D) PCR-RFLP analysis of TNF-α −863 C/A polymorphism on 10% polyacrylamide gel: lanes: 1, 3, 4, 5 & 6 show homozygous (CC) genotypes; lane: 2 shows heterozygous (CA) genotype; lane M shows 100 bp DNA ladder. (E) PCR-RFLP analysis of TNF-α −1031 T/C polymorphism on 2.0% agarose gel: lanes: 4 & 5 show homozygous (TT) genotypes; lanes: 6 & 7 show heterozygous (TC) genotypes; lanes: 1, 2 & 3 show homozygous (CC) genotype; lane M shows 100 bp DNA ladder. (TIF) [file pone.0052298.s001.tif]

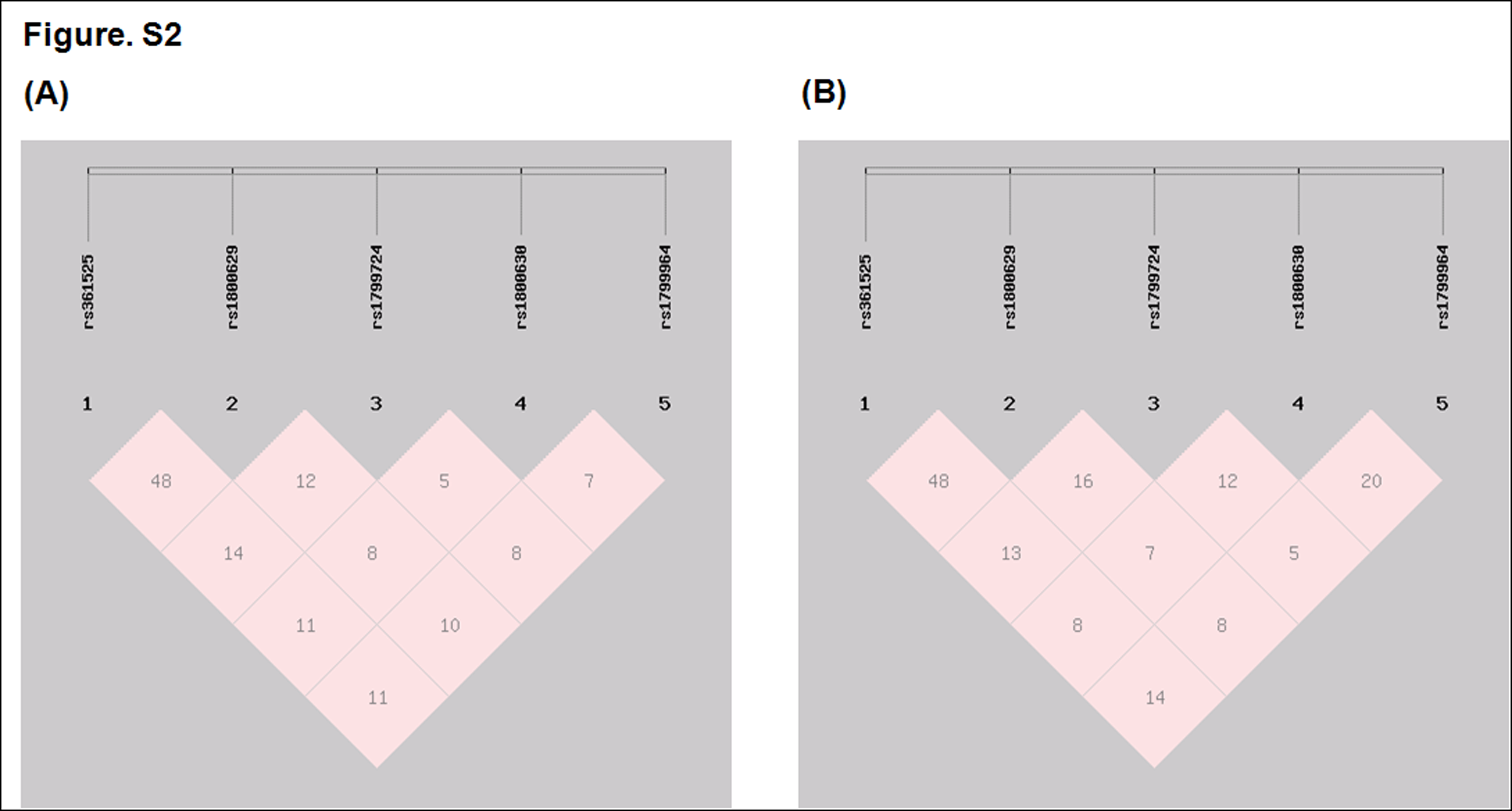

Supplement: Figure S2 — Linkage disequilibrium analysis of TNF -α promoter polymorphisms. (A) Linkage disequilibrium (D’) among TNF-α promoter SNPs in generalized vitiligo patients and controls from Gujarat population. (B) Linkage disequilibrium (D’) among TNF-α promoter SNPs in localized vitiligo patients and controls from Gujarat population. (TIF) [file pone.0052298.s002.tif]

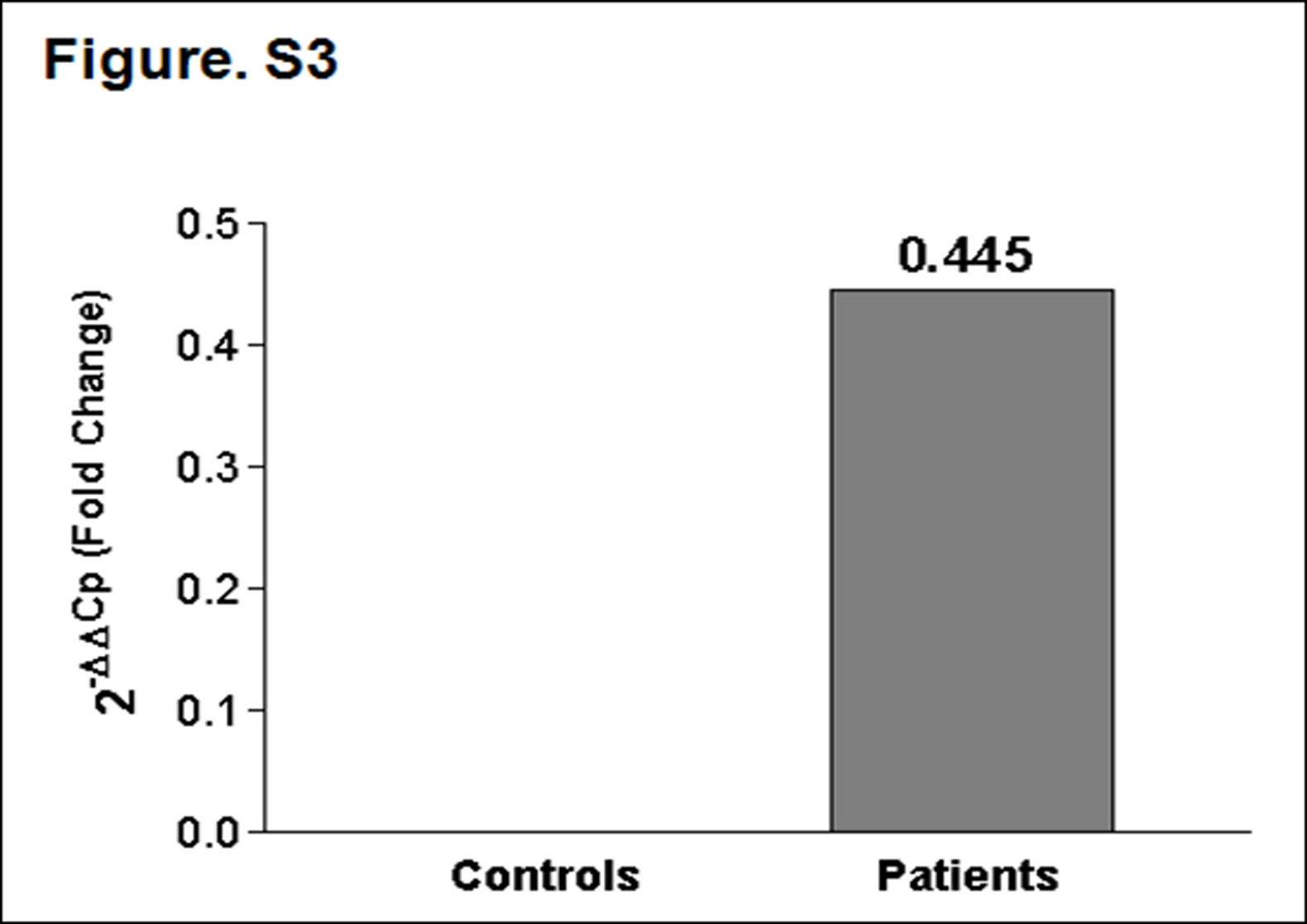

Supplement: Figure S3 — Expression fold change of TNF -α transcript in 157 vitiligo patients against 174 controls showed 0.445 fold change as determined by 2− ΔΔCp method. (TIF) [file pone.0052298.s003.tif]
