# Supplementary material for: Increased Tumor Necrosis Factor (TNF)-α and Its Promoter Polymorphisms Correlate with Disease Progression and Higher Susceptibility towards Vitiligo
Source: PLoS One. 2012 Dec 20;7(12):e52298. doi: 10.1371/journal.pone.0052298 (PMC3527546; doi:10.1371/journal.pone.0052298)
Supplement: Table S1 — Demographic characteristics of vitiligo patients and unaffected controls of Gujarat. (DOC) [file pone.0052298.s004.doc]

**Table S1.** Demographic characteristics of vitiligo patients and unaffected controls of Gujarat.

|  | **Vitiligo Patients** |  | **Controls** |
| --- | --- | --- | --- |
| Average age  (mean age ± SD)  Sex: male  female  Age of onset  (mean age ± SD)  Duration of disease  (mean ± SD)  Type of vitiligo  Generalized  Localized  Active vitiligo  Stable vitiligo | (n = 977) |  | (n = 990) |
|  | 32.45 ± 13.48 yr  451 (46.16%)  526 (53.84%)  21.25 ± 12.53 yr  7.8 ± 6.9 yr  733 (75.03%)  244 (24.97%)  682 (69.81%)  295 (30.19%) |  | 28.23 ± 14.42 yr  447 (45.15%)  543 (54.85%)  NA  NA  NA  NA  NA  NA |
