# Supplementary material for: Increased Tumor Necrosis Factor (TNF)-α and Its Promoter Polymorphisms Correlate with Disease Progression and Higher Susceptibility towards Vitiligo
Source: PLoS One. 2012 Dec 20;7(12):e52298. doi: 10.1371/journal.pone.0052298 (PMC3527546; doi:10.1371/journal.pone.0052298)
Supplement: Table S2 — Primers used for TNF -α promoter SNPs genotyping and gene expression analysis. (DOC) [file pone.0052298.s005.doc]

**S2.** Primers used for *TNF*-α promoter SNPs genotyping and gene expression analysis.

| **Gene/SNP**^*^ **Primer** | **Sequence (5’ to 3’)** | **Annealing**  **Temp.**  **(°C)** | **Product**  **size**  **(bp)** | **Restriction**  **Enzyme**  **(Digested**  **Products)** |
| --- | --- | --- | --- | --- |
| **(rs361525)**  *TNF*-α -238G/A F  *TNF*-α -238G/A R  **(rs1800629)**  *TNF*-α -308G/A F  *TNF*-α -308G/A R  **(rs1799724)**  *TNF*-α -857C/T F  *TNF*-α -857C/T R  **(rs1800630)**  *TNF*-α -863C/A F  *TNF*-α -863C/A R  **(rs1799964)**  *TNF*-α -1031T/C F  *TNF*-α -1031T/C R  *TNF*-α gene expression F    *TNF*-α gene expression R  *GAPDH* gene expression F  *GAPDH* gene expression R | CTGTCCCAGGCTTGTCCTGCTAC  CTCACACTCCCCATCCTCCC**G**G**A**TC  GAGGCAATAGGTTTTGAGGG**C**CAT  TCTGCTGTCCTTGCTGAGGGA  GCATCTGCACCCTCGATGAAG  CCTCTACATGGCCCTGTCTAC  GCTCAAAGGGAGCAAGAGCTG  CTACATGGCCCTGTCTTCGTTACG  GCTCAAAGGGAGCAAGAGCTG  GCTGGTTTCAGTCTTGGCTTCC  GCCCCCAGAGGGAAGAGTTCCCCA  GCT TGAGGGTTTGCTACAACATGGGC  CATCACCATCTTCCAGGAGCGAG  CCTGCAAATGAGCCCCAGCCT | 66  57  58  65  66  65  65 | 376    360    325    323    481    124    122 | *Bam*HI  (352bp & 24bp)      *Nco*I  (339bp & 21bp)    *Tai*I  (306bp & 19bp)    *Tai*I  (302bp & 21 bp)      *Bbs*I  (313bp & 168bp)  -    - |
|  |  |  |  |

*The nucleotide change was from the ancestral (major) to the derived (minor) allele.

Bold letters within the primer sequences represent a forced mismatch.
