# Supplementary material for: Increased Tumor Necrosis Factor (TNF)-α and Its Promoter Polymorphisms Correlate with Disease Progression and Higher Susceptibility towards Vitiligo
Source: PLoS One. 2012 Dec 20;7(12):e52298. doi: 10.1371/journal.pone.0052298 (PMC3527546; doi:10.1371/journal.pone.0052298)
Supplement: Table S4 — Pairwise linkage disequilibrium (D’) values between TNF -α SNPs with >3% minor allele frequencies within localized vitiligo patients and controls from Gujarat population. (DOC) [file pone.0052298.s007.doc]

**Table S4.** Pairwise linkage disequilibrium (D’) values between *TNF*-α SNPs with >3% minor allele frequencies within localized vitiligo patients and controls from Gujarat population.

|  | rs1800629  (-308 G/A) | rs1799724  (-857 C/T) | rs1800630  (-863 C/A) | rs1799964  -1031 T/C |
| --- | --- | --- | --- | --- |
| rs361525  (-238 G/A) | **0.484** | 0.131 | 0.083 | 0.135 |
| rs1800629  (-308 G/A) | - | 0.159 | 0.073 | 0.076 |
| rs1799724  (-857 C/T) | - | - | 0.123 | 0.052 |
| rs1800630  (-863 C/A) | - | - | - | 0.200 |

Bold value represents moderate LD.
